# The History and Evolution of the Stethoscope

**DOI:** 10.7759/cureus.28171

**Published:** 2022-08-19

**Authors:** Misha Choudry, Thor S Stead, Rohan K Mangal, Latha Ganti

**Affiliations:** 1 Biology, Trinity Preparatory School, Orlando, USA; 2 Medicine, The Warren Alpert Medical School of Brown University, Providence, USA; 3 Medicine, University of Miami Miller School of Medicine, Miami, USA; 4 Emergency Medicine, Envision Physician Services, Plantation, USA; 5 Emergency Medicine, University of Central Florida College of Medicine, Orlando, USA; 6 Emergency Medicine, HCA Ocala Hospital, Ocala, USA

**Keywords:** ebers papyrus, medical tools, cardiology devices, medicine history, stethoscope

## Abstract

This paper is a summary of the evolution of the stethoscope. It goes through the major stages of stethoscope evolution, starting with the first recorded breath sounds and going all the way to the most recent, entirely automated stethoscope pads. The iconic stethoscope has undergone many changes and evolved with the times to earn its place slung around the neck of a physician. This review traces its journey.

## Introduction and background

**Figure 1:**
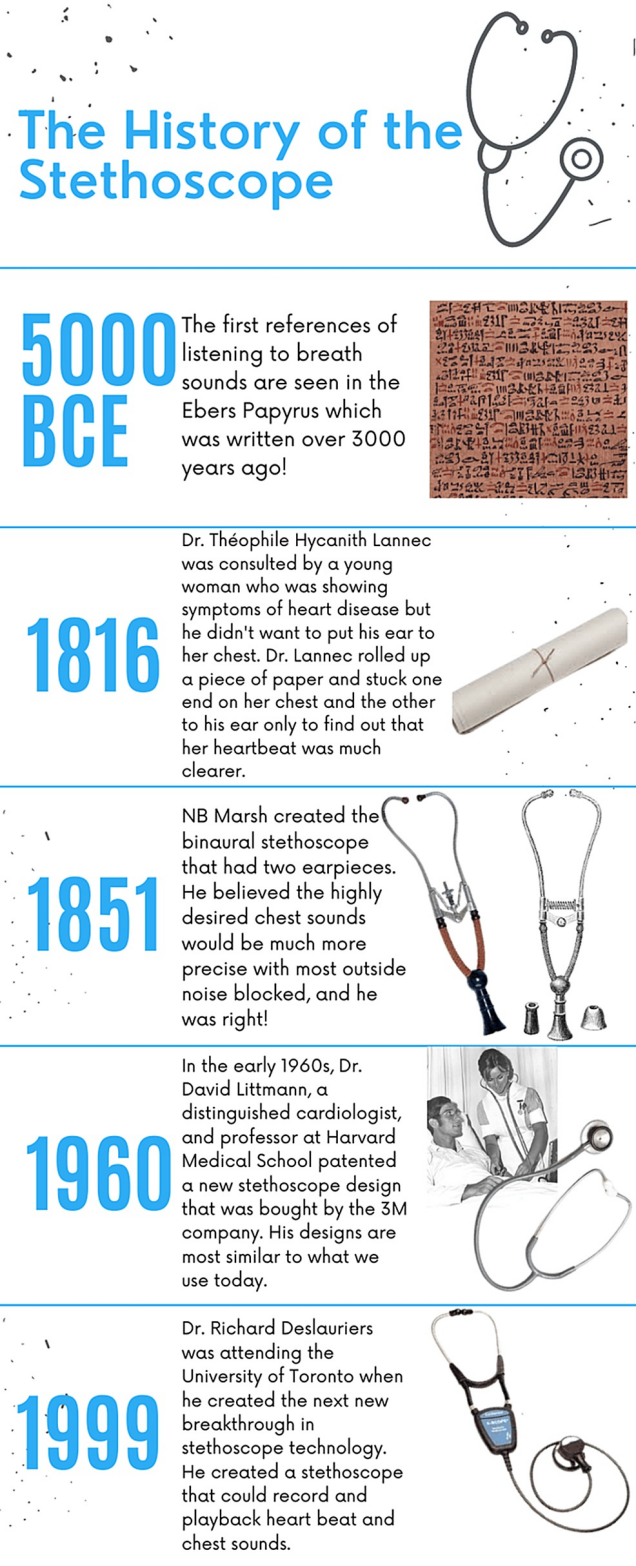
A timeline of the major improvements of the stethoscope. All images are from free sources such as creative commons and Getty images.

The stethoscope is the image of medicine. It is a visible sign of the years of education a doctor went through to receive their title and their ability to practice. It is an iconic and commonly used tool, and its importance in the field is immeasurable. It is very difficult to fathom what medicine was like pre-stethoscope and how anything got done without the tool is miraculous. In order to earn its place slung around the neck of a physician, the stethoscope has undergone many changes and evolved with the times. Like all aspects of medicine, the stethoscope has a long history and background (Figure 1).

## Review

The first reference to listening to breath sounds was in the Ebbers Papyrus in 1,500 BCE (Before the Common Era). That was almost 4,000 years ago. Some other early cases of listening to breath sounds are recorded in the Hindu Vedas from approximately 1,400 to 1,200 BCE and in the Hippocratic Writings from approximately 440 to 360 BCE [1].

There are two versions of the birth of the stethoscope; the first is what is taught today. In the first and more likely version, most sources cite the first use of a tool to be used to hear heartbeats and chest sounds in 1816 by Dr. Théophile Hycanith Laennec. In the more widely spread version of the story, Dr. Lannec was consulted by an overweight young woman who was showing symptoms of heart disease. Due to her size and age, Dr. Lannec felt it was improper to put his head on her chest to listen to her heart. Despite this being common practice at the time, it was not very accurate and often led to misdiagnosis and even no diagnosis at all! To listen to the young lady's heart, he rolled up a piece of paper into a tube. He placed one end of the tube on the precordial region and in the other, he placed his ear. He discovered that the tube and its direct application to the chest made both breath sounds and heart sounds much clearer. The acoustics of the paper tube was not perfect, but they were noticeably better than the direct ear-to-chest listening that was deemed the best option at the time [2]. In the second version (which is much less likely but much more entertaining), Dr. Lannec had just come home to his wife in a very bad mood. She had heard at her sewing circle that he was touching and putting his head on young women’s chests, and she had been terribly embarrassed. Despite the doctor explaining that it was his job, Mrs. Lannec refused to hear him out. So, to combat this, Dr. Lannec took his wife's words into account and created a cone-shaped piece of paper. After discovering how effective it was, he carved a new tool with an ivory tip on both ends to conduct heat. The tool was later used to listen to the fetus inside a pregnant woman because it was found to be more accurate and powerful [3].

By the 1820s, the stethoscope was readily available all over Europe, and doctors were experimenting with different sizes, shapes, and materials in order to create the most effective tool. In 1851, the binaural stethoscope was invented by NB Marsh. He believed that the highly desired chest sounds would be much more precise with most outside noise blocked, and he was right! As time passed, stethoscopes had variations based on what they were going to be used for. Doctors who dealt heavily with patients who had contracted highly contagious illnesses were known to have used stethoscopes that were up to 35 cm (13.5 inches) long to keep a distance. Along with that, special stethoscopes were designed for children that were smaller and shorter. Rubber was introduced to the general public in 1853, and the stethoscope evolved from a cone- or horn-shaped brass instrument to one with ivory earpieces, a wooden chest piece, and wooden tubing held together by rubber bands [4,5].

In the early 1960s, Dr. David Littmann, a distinguished cardiologist and professor at Harvard Medical School, patented a new stethoscope design that was bought by the 3M company. Spinoffs of Dr. Littmann's design are most similar to what is used today in modern medicine. In the late 1970s, the 3M-Littmann company introduced a tunable diaphragm. In a stethoscope, a diaphragm is a thin sheet of material that forms a partition. It is used in acoustic systems to get the best sound possible. The new stethoscope had a very hard epoxy resin-glass diaphragm member with over-molded silicon. The flexible acoustic surround made it much easier to collect sound. The sound was clear and tangible for even the most inexperienced stethoscope users to hear [6-8].

The next big development in stethoscope technology would come about in 1999. Dr. Richard Deslauriers was attending the University of Toronto intending to invent new medical devices, despite having spent five years in medical school developing the stethoscope, with all of his work funded by his work for Johnson & Johnson. The new stethoscope was called a recording stethoscope and was able to record and playback chest sounds and heartbeats. All the acoustic technology was housed in a chest piece that was the diameter of a silver dollar [6-8]. During the development of the stethoscope, Dr. Deslauriers worked closely with engineers from the Bose Corporation (a company that makes speakers and headphones). While working with the company, Dr. Deslauriers created a specially insulated tubing to block out sounds like the stethoscope rubbing against a shirt. This new recording stethoscope was groundbreaking. Recordings of things like chest sounds could be added to medical records and played back to listen to abnormalities in a way that simply was not accessible. The only downside to this new stethoscope was the relatively expensive price. It is not yet accessible to all doctors, hospitals, and clinics, but is sure to be in the near future [9].

But as we journey further into the 20th century, some doctors have decided that stethoscopes are becoming obsolete. During the early stages of the COVID-19 pandemic, doctors had a hard time hearing chest and breath sounds due to how highly contagious COVID-19 was. This was quite problematic because COVID-19 was a respiratory illness, and changes in breath sounds can provide vital information in patients with respiratory disease. To combat this, many doctors use ultrasounds for heartbeat monitoring and to monitor breathing [10-12]. Biochemical engineers have been working on an even more efficient type of stethoscope that is very different from what we are used to. It is a foam pad that can simultaneously auscultate 14 chest wall locations using 14 super powerful embedded microphones. The new tool is very accurate, and in a trial of 100 pneumonia patients, they found that 91% of the patients had adventitious lung sounds, with 89% having crackle sounds and 63% having a high-or low-pitched wheeze [13-15]. Although this new system may seem perfect, it takes two minutes per microphone. This means that getting simple chest sounds would take 28 minutes. Despite this limitation, this breakthrough exemplifies the continued evolution of the stethoscope.

## Conclusions

The stethoscope has taken its place in the medical hall of fame, and its spot is well deserved. What started as a rolled-up piece of paper has become a tool of immeasurable value. Over the course of 300 years, the stethoscope has evolved from a paper tube to a horn-shaped instrument, to a binaural stethoscope, to the Littmann stethoscope, and finally to the recording and electronic stethoscopes. Even today, the stethoscope is still evolving and growing to eventually get to its most efficient, effective, and accurate form. We can only hope to one day see the epitome of medical technology in the best stethoscope.
